# Presymptomatic Treatment With Andrographolide Improves Brain Metabolic Markers and Cognitive Behavior in a Model of Early-Onset Alzheimer’s Disease

**DOI:** 10.3389/fncel.2019.00295

**Published:** 2019-07-18

**Authors:** Pedro Cisternas, Carolina A. Oliva, Viviana I. Torres, Daniela P. Barrera, Nibaldo C. Inestrosa

**Affiliations:** ^1^Centro de Envejecimiento y Regeneración, Departamento de Biología Celular y Molecular, Facultad de Ciencias Biológicas, Pontificia Universidad Católica de Chile, Santiago, Chile; ^2^Centre for Healthy Brain Ageing, School of Psychiatry, Faculty of Medicine, University of New South Wales, Sydney, NSW, Australia; ^3^Centro de Excelencia en Biomedicina de Magallanes, Universidad de Magallanes, Punta Arenas, Chile

**Keywords:** andrographolide, Wnt signaling, glucose metabolism, Alzheimer’s disease, neuroprotection

## Abstract

Alzheimer’s disease (AD) is the most common type of dementia. The onset and progression of this pathology are correlated with several changes in the brain, including the formation of extracellular aggregates of amyloid-beta (Aβ) peptide and the intracellular accumulation of hyperphosphorylated tau protein. In addition, dysregulated neuronal plasticity, synapse loss, and a reduction in cellular energy metabolism have also been described. Canonical Wnt signaling has also been shown to be downregulated in AD. Remarkably, we showed previously that the *in vivo* inhibition of Wnt signaling accelerates the appearance of AD markers in transgenic (Tg) and wild-type (WT) mice. Additionally, we found that Wnt signaling stimulates energy metabolism, which is critical for the ability of Wnt to promote the recovery of cognitive function in AD. Therefore, we hypothesized that activation of canonical Wnt signaling in a presymptomatic transgenic animal model of AD would improve some symptoms. To explore the latter, we used a transgenic mouse model (J20 Tg) with mild AD phenotype expression (high levels of amyloid aggregates) and studied the effect of *andrographolide* (ANDRO), an activator of canonical Wnt signaling. We found that presymptomatic administration of ANDRO in J20 Tg mice prevented the reduction in cellular energy metabolism markers. Moreover, treated animals showed improvement in cognitive performance. At the synaptic level, J20 Tg animals showed severe deficiencies in presynaptic function as determined by electrophysiological parameters, all of which were completely restored to normal by ANDRO administration. Finally, an analysis of hippocampal synaptosomes by electron microscopy revealed that the length of synapses was restored with ANDRO treatment. Altogether, these data support the idea that the activation of canonical Wnt signaling during presymptomatic stages could represent an interesting pharmacological strategy to delay the onset of AD.

## Introduction

Described over 100 years ago, Alzheimer’s disease (AD) has become the most common age-related neurodegenerative disorder. AD is mainly characterized by progressive cognitive impairment, and current therapeutic approaches are limited by uncertainties about its precise etiology (Serrano-Pozo et al., 2011; Wang et al., 2017). This pathology is characterized by a slow loss of learning and memory cognitive functions, concomitant with the presence of senile plaques (Aβ aggregates) and neurofibrillary tangles in the hippocampus and cerebral cortex (Querfurth and LaFerla, 2010; Serrano-Pozo et al., 2011). Other molecular events also related to the progression of the disease include an increase in reactive oxygen species (ROS) production, mitochondrial dysfunction, inflammation, synaptic loss and a decrease in cerebral glucose uptake/utilization (Hamos et al., 1989; Kapogiannis and Mattson, 2011; Chen and Zhong, 2013; Cisternas and Inestrosa, 2017). Recently, the dysregulation of glucose metabolism has been postulated as one of the first events of AD pathology (Cisternas and Inestrosa, 2017). Early modulation of glucose metabolism has become an attractive target for AD patients in whom cognitive impairment has not yet become evident. Indeed, it has been documented that administration of hormones that stimulate glucose metabolism improves cognitive responses in humans diagnosed with AD, as well as in murine models of AD (Reger et al., 2006; Craft et al., 2012; Chapman et al., 2013; Freiherr et al., 2013). These findings suggest that the dysregulation of glucose in the brain could be critical for the onset and progression of AD and the development of other metabolic diseases, including diabetes.

Since the discovery of Wnt signaling three decades ago, this pathway has been implicated in several processes during the development and maintenance of the adult central nervous system (CNS). These processes include neurogenesis, neurite outgrowth, synapse establishment and maturation, and synaptic function (Ciani and Salinas, 2005; Varela-Nallar et al., 2010; Oliva et al., 2018). A strong relationship between loss of Wnt signaling function and neuronal dysfunction in AD has been documented (De Ferrari et al., 2003; Ciani and Salinas, 2005; Toledo and Inestrosa, 2010; Budnik and Salinas, 2011; Oliva et al., 2013). For example, in patients with AD, a decrease in the levels of canonical Wnt effectors such as β-catenin and an increase in the levels of Wnt inhibitors such as Dkk1 have been described (Purro et al., 2012, 2014). On the other hand, the presence of Aβ triggers a decrease in Wnt signaling in animal models of the disease (De Ferrari et al., 2003; Alvarez et al., 2004; Caricasole, 2004; Inestrosa et al., 2012). In a recent study, we showed that the inactivation of Wnt signaling in a transgenic mouse model of AD accelerates the progression of the disease. Specifically, we found increases in cognitive deficits, tau phosphorylation, and the Aβ_42_/Aβ_40_ ratio, as well as high levels of soluble Aβ species and changes in the number and size of senile plaques. Similarly, the inactivation of canonical Wnt signaling in WT mice triggers an increase in tau phosphorylation and hippocampal dysfunction, which correlates with the increase in Aβ_1-42_ levels (Tapia-Rojas and Inestrosa, 2018). Altogether, the evidence suggests that Wnt signaling stability is required to guarantee a normal phenotype.

From previous data, we know that canonical and non-canonical Wnt signaling stimulate glucose metabolism in cultured hippocampal neurons by promoting glucose uptake and utilization (Cisternas et al., 2016a,b). Additionally, *in vivo* studies have demonstrated that symptomatic administration of canonical Wnt signaling activators (ANDRO and lithium) in a Tg AD mouse rescue the cognitive performance of the animals (Cisternas et al., 2018). This effect is mediated by an improvement of cellular glucose metabolism through a mechanism partially dependent on canonical Wnt signaling (Cisternas et al., 2018). Here, we provide evidence that the administration of the canonical Wnt signaling activator ANDRO, starting in the presymptomatic stage in J20 Tg mice, is able to rescue several cellular and physiological parameters. ANDRO improves cellular glucose metabolism and cognitive performance, both of which are accompanied by the recovery of neuronal aspects related to synaptic plasticity and morphology.

## Materials and Methods

### Animals and Ethical Standards

High accumulation of amyloid aggregates, a condition described in humans with AD, was modeled in the PPSwInd Tg mouse line, which expresses a mutant form of human amyloid precursor protein (APP) bearing both the Swedish (K670N/M671L) and Indiana (V717F) mutations (APPSwInd). This mouse line is known as J20 and was obtained from The Jackson Laboratory (Stock no. 004462; Bar Harbor, ME, United States; RRID: MMRRC_034829-JAX). Three-month-old male (Hemizygous) mice were used as an asymptomatic AD model before starting the treatment (Mucke et al., 2000). Male C57BL/6 WT mice (3 months old) were used as a control.

The animals were maintained at the Animal Facility of the Pontifical Universidad Católica de Chile behind sanitary barriers in ventilated racks and in closed colonies. No sample-size calculation was performed. The experimental procedures were approved by the Bioethical and Biosafety Committee of the Faculty of Biological Sciences of the Pontifical Universidad Católica de Chile. Thirty mice were used and handled according to the National Institutes of Health guidelines (NIH, Baltimore, MD, United States). We used simple randomization to allocate mice to different cages. We divided the J20 animals into two groups (10 animals per group). In one group, we administered andrographolide (CAS Number 5508-58-7, cat: 365645, ANDRO, 2 mg/kg) three times per week for 16 weeks by intraperitoneal injection. The second group of J20 and the WT mice (10 animals each) were injected with saline solution as vehicle for 16 weeks. After the treatment, seven animals per group were used for the cognitive performance tests. After the cognitive test, the animals were housed for another 7 days and then used in the electrophysiology recording and other experiments. Only animals that completed the entire treatment and appeared healthy were used for the studies. For the inclusion/exclusion criterion, we evaluated the weight, and a visual inspection was performed. To prevent animal suffering, technical personnel checked the animals daily for evidence of distress (NIH tables of supervision). All the cognitive tests (training and experiments) and the slice experiments were performed in a double-blind manner.

### D-[1-^14^C] Glucose Biodistribution

Upon completing the cognitive tests, three mice from each group were injected with D-[1-^14^C] glucose (cat: NEC043X001 MC, PerkinElmer, United States) via the tail vein. Briefly, mice were anesthetized with isoflurane and injected intravenously via the tail with 50 μCi of tracer diluted to a final volume of 20 μL in isotonic saline. Following a 15 min uptake period, the animals were killed, and tissues were collected. Tissue radioactivity was quantified by liquid scintillation. D-[1-^14^C] glucose levels were normalized to the weight of resected tissue and expressed as the percent of injected dose (Tsytsarev et al., 2012; Cox et al., 2015).

### Glucose Uptake Analysis

Hippocampal slices were washed with buffer (15 mM HEPES (cat: H3375), 135 mM NaCl (cat: s3014), 5 mM KCl (cat: P5405), 1.8 mM CaCl_2_ (cat: C1016), and 0.8 mM MgCl_2_ (cat: 208337) supplemented with 0.5 mM glucose (Cisternas et al., 2014a). Then, the slices were incubated for 0–90 min with 1–1.2 μCi 2-[1,2-^3^H(N)]-deoxy-D-glucose (cat: NET328250UC, PerkinElmer, United States) or ^3^H-2-deoxyglucose (cat: NET328A250UC, PerkinElmer, United States) at a final specific activity of 1–3 disintegrations/min/pmol (∼1 mCi/mmol). Glucose uptake was arrested by washing the cells with ice-cold PBS supplemented with 1 mM HgCl_2_ (cat: 203777, Sigma-Aldrich, United States). The incorporated radioactivity was quantified by liquid scintillation counting.

### Determination of the Glycolytic Rate

Glycolytic rates were determined as previously described (Cisternas et al., 2014a). Briefly, slices were placed in tubes containing 5 mM glucose and then washed twice in Krebs–Henseleit solution (11 mM Na_2_HPO_4_, 122 mM NaCl, 3.1 mM KCl, 0.4 mM KH_2_PO_4_, 1.2 mM MgSO_4_, and 1.3 mM CaCl_2_, pH 7.4) containing the appropriate concentration of glucose. After equilibration in 0.5 mL of Hank’s balanced salt solution/glucose (cat: 14025076, Thermo Fisher, United States) at 37°C for 30 min, 0.5 mL of Hank’s balanced salt solution containing various concentrations of [3-^3^H] glucose (cat: NET331C250UC, PerkinElmer, United States) was added, with a final specific activity of 1–3 disintegrations/min/pmol (∼1 mCi/mmol). Aliquots of 100 μL were then transferred to another tube, placed inside a capped scintillation vial containing 0.5 mL of water, and incubated at 45°C for 48 h. After this vapor-phase equilibration step, the tube was removed from the vial, the scintillation mixture was added, and the ^3^H_2_O content was determined by counting over a 5-min period.

### Quantification of Hexokinase (HK) Activity

Brain tissue was washed with PBS, treated with trypsin/EDTA, and centrifuged at 500 × *g* for 5 min at 4°C. Then, the tissue was resuspended in isolation medium (250 mM sucrose (cat: S9378), 20 mM HEPES, 10 mM KCl, 1.5 mM MgCl_2_, 1 mM EDTA (cat: E6758), 1 mM DTT (cat: D0632), 2 mg/mL aprotinin (cat: A1153), 1 mg/mL pepstatin A (cat: 77170), and 2 mg/mL leupeptin (cat: L8511) at a 1:3 dilution, sonicated at 4°C, and then centrifuged at 1,500 × *g* for 5 min at 4°C. HK activity of the supernatant was quantified. For the assay, the purified fraction was mixed with the reaction medium (25 mM Tris-HCl (cat: T5941), 1 mM DTT, 0.5 mM NADP/Na^+^ (cat: N8035), 2 mM MgCl_2_, 1 mM ATP (cat: A1852), 2 U/mL G6PDH (cat: G6378), and 10 mM glucose (cat: G8270), and the mixture was incubated at 37°C for 30 min. The reaction was stopped by the addition of 10% trichloroacetic acid (cat: T6399, TCA), and the generation of NADPH was measured at 340 nm (Cisternas et al., 2016a). All reagents were purchased from Sigma-Aldrich, United States.

### ATP Content

Brain tissues or hippocampal slices were treated with activators/inhibitors, and ATP levels were measured using an ATP determination kit (cat: A22066, Invitrogen/Molecular Probes, United States) (Calkins et al., 2011).

### Activity of PFK and AMPK

For brain samples and hippocampal slices, AMPK activity was measured using an antibody specific to the phosphorylated T-172 (active) form of AMPK-α. Detection was performed by ELISA following the manufacturer’s protocol. Experiments were conducted in triplicate and repeated at least three times (cat: KHO0651, Thermo Fisher Scientific Inc., United States) (Moreno-Navarrete et al., 2011; Stow et al., 2016). PFK activity was measured using the PFK Colorimetric Assay Kit (cat: K776, BioVision, United States) according to the manufacturer’s instructions (Thurley et al., 2017).

### Large Open-Field (LOF) Test

A 120 × 120 cm transparent Plexiglas cage with 35-cm-high transparent walls was used to study locomotor and stress behavior in our mouse model. The open field, which measured 40 × 40 cm, was defined as the “center” area of the field. Data were collected using an automatic tracking system (HVS Imagen, United Kingdom). Each mouse was placed alone in the center of the open field, and its behavior was tracked for 20 min. At the end of the session, the mouse was returned to its home cage. The parameters measured included the total time moving and the number of times the mouse crossed the center area of the platform (Walsh and Cummins, 1976; Cisternas et al., 2015).

### Novel Object Recognition (NOR) and Novel Object Location (NOL)

The novel object recognition (NOR) and novel object location (NOL) tasks were performed as previously described (Bevins and Besheer, 2006; Vargas et al., 2014). Mice were habituated to the experimental room in the experimental cages for three consecutive days for 30 min per day (three consecutive days) and for 1 h on the testing day. The task was conducted on a 120 × 120 cm transparent Plexiglas platform with 35-cm-high transparent walls containing two identical objects placed at specific locations. For object familiarization, mice were allowed to explore the platform for 10 min. The animals were subsequently returned to their home cages for 1 h, followed by a 5 min exposure to a novel localization of one of the familiar objects (NOL). The mice were again returned to their home cages for 1 h and were subsequently exposed to a novel object for 5 min. The mice had no observed baseline preference among the different objects. An object preference index was determined by calculating the time spent near the relocated/novel object divided by the cumulative time spent with both the familiar and relocated/novel objects. The cages were routinely cleaned with ethanol following mouse habituation and testing.

### Memory Flexibility Test

This test was performed as previously described, and the conditions of the pool were the same as those of a Morris Water Maze (Chen et al., 2000; Salazar et al., 2017). Each animal was trained to one pseudorandom location of the platform per day for 5 days, with a new platform location each day. Training was conducted for up to 10 trials per day until the criterion of three successive trials with an escape latency of 20 s was achieved. When testing was completed, the mouse was removed from the maze, dried and returned to its cage. Animals were tested for the next location on the following day. Data were collected using a video tracking system (HVS Imagen).

### ADP Content

ADP levels in whole-cell lysates of primary neurons and slices were measured using an ADP Assay Kit (cat: ab83359, Abcam, United Kingdom), according to the manufacturer’s instructions (Chen et al., 2000).

### Preparation of Mouse Brain Slices for Electrophysiology

Male mice at the age of 7 months were used to prepare acute coronal slices. Each animal was anesthetized with isoflurane and killed by decapitation, and the brain was removed rapidly. The brain was placed in a beaker with a cold sucrose-based solution composed of the following (in mM): 85 NaCl, 75 sucrose, 3 KCl, 1.25 NaH_2_PO_4_, 25 NaHCO_3_, 10 dextrose, 3.5 MgSO_4_, 0.5 CaCl_2_, 3 sodium pyruvate, 0.5 sodium L-ascorbate and 3 myo-inositol (305 mOsm, pH 7.4). Coronal sections (350 μm) were cut with a vibratome, and every slice was transferred to a beaker containing the same solution but at 36°C. An hour later, this solution was changed to recording solution, composed of (in mM): 126 NaCl, 3.5 KCl, 1.25 NaH_2_PO_4_, 25 NaHCO_3_, 10 dextrose, 1 MgSO_4_, 2 CaCl_2_, 3 sodium pyruvate, 0.5 sodium L-ascorbate and 3 myo-inositol (305 mOsm, pH 7.4) at room temperature (22°C). The slices were maintained there until used for recording. The recording was performed in the same solution, but its temperature was increased to 32–34°C for the full duration of the experiment. To prevent the γ-aminobutyric acid (GABA_A_)-mediated inhibitory component from interfering with excitatory postsynaptic potentials (EPSPs), we applied 25 μM of picrotoxin. The experimental data were obtained from at least two brain slices from each animal. Multiple slices from a single animal were considered replicates. They were subsequently averaged representing a single animal. Hence, for WT *n* = 8 mice, two slices each; for J20 Tg: *n* = 6 mice, three slices each; and for J20+ANDRO: *n* = 6 mice, three slices each. Finally, all the n_s_ values in every experimental group (WT, J20 or J20+ANDRO) were also averaged and expressed as mean ± SEM.

### Electrophysiological Recordings and Data Analysis

The slices were placed in a chamber under an upright infrared-differential interference contrast (IR-DIC) fluorescence microscope (Eclipse FNI, Nikon) equipped with a 40× water objective and a light-sensitive camera (TOPICA CCD Camera). We stimulated the Schaffer collaterals between CA3 and CA1 with a bipolar concentric electrode (World Precision Instruments, Sarasota, FL, United States) connected to an ISO-Flex stimulus generator (A.M.P.I., Jerusalem, Israel). To record the evoked field excitatory postsynaptic potentials (fEPSPs), we used a borosilicate glass electrode (World Precision Instruments, United States) ranging from 0.5 to 1 MΩ pulled on a P-97 Flaming/Brown Micropipette Puller (Sutter Instruments, United States). Each glass pipette was filled with the recording solution and placed in the *stratum radiatum* of the CA1 hippocampal region. The signal was collected using a MultiClamp 700B amplifier (Axon CNS, Molecular Devices LLC, United States) and digitally sampled at 30 kHz using a Digidata-1440A interface (Axon CNS, Molecular Devices). The data were acquired and analyzed offline using pClamp 10 (Molecular Devices LLC, United States). First, in each slice, we determined the relationship between the stimulus intensity of input and the magnitude of the evoked response. For this purpose, increasing levels of current were applied, and the slope of evoked fEPSP was determined. The intensity to evoke 60% of the maximum amplitude was used to perform the experiments. Second, to calculate the paired-pulse facilitation (PPF) index, we calculated the ratio of the second EPSP slope (R2) by the first EPSP slope (R1), evoked at different interstimulus intervals between 10 and 200 ms. Third, for long-term plasticity, every experiment consisted of the application of two pulses separated 50 ms each, every 15 s. The slope value of the first pulse (R1) was averaged during 15 to 20 min before TBS to obtain basal data, and then during 60 min after TBS to obtain the LTP induced data. We allowed enough time to obtain a stable basal signal and have at least 20 min of continuous data before applying theta burst stimulation (TBS). The necessary time period consisted of a series of 5 burst at 100 Hz, every 20 s. Subsequently, the same two pulses every 15 s were taken for 60 min more. To calculate the amount of long-term potentiation (LTP), we measured the slope (mV/ms) of the first fEPSP and averaged the 20 min pre-TBS and normalized pre- and post-TBS against this value (mean) at each time point. The normalized change was plotted as the relative value of fEPSP slope. To determine the degree of potentiation among groups, we compared. We also measured the fiber volley (FV) amplitude to show the correlation with fEPSP slope changes. Further analyses were performed using the same data. To determine the degree of facilitation, we measured the slope of the first evoked fEPSP (R1) and the slope of the second evoked fEPSP (R2), and we established the (R2 - R1)/R1 or relative facilitation, which is a measure of facilitation, before and after TBS. These data were used to determine the PPF index, which is a measure of presynaptic Ca^2+^ and PPF attenuation, which correlates the PPF with LTP and postsynaptic mechanisms. All values of synaptic responses are presented as the mean ± SEM.

### Synaptosome Preparation

Synaptosomes were prepared from hippocampus samples isolated from mouse brains. Hippocampi were homogenized in a weight:volume ratio of 1:10 using cold lysis buffer containing 0.3 M sucrose, 5 mM Hepes (pH 7.4) and protease and phosphatase inhibitors using an automatic Potter Elvehjem tissue homogenizer with Teflon Pestle (Pyrex) for 10 strokes at 3,000 rpm. The homogenate was centrifuged at 1,000 × *g* for 20 min, and then the supernatant was centrifuged at 16,000 × *g* for 20 min. The resultant pellet was resuspended in buffer 0.32 M sucrose, 5 mM Hepes pH 7.4 and passed six times through a 21-gauge syringe. The resuspended pellet was layered on top of a sucrose gradient of 0.85 M, 1.0 M, and 1.2 M and centrifuged at 100,000 × *g* for 2 h. The interface between 1.0 M and 1.2 M sucrose, corresponding to enriched synaptosomes, was fixed for electron microscopy. Synaptosomes were prepared from three animals in each group, and five to eight synapses were analyzed per group. Tukey’s multiple comparison test was performed to compare the means.

### Electron Microscopy

The synaptosome pellet was fixed with 2.5% glutaraldehyde in 0.1 M sodium cacodylate buffer pH 7.0 at room temperature overnight and was washed in three changes of cacodylate buffer for 2 h. The pellet was postfixed with aqueous 1% osmium tetroxide for 120 min, and after a rinse with bidistilled water, it was stained in block with 1% uranyl acetate for 90 min. The specimen was then dehydrated with graded concentrations of acetone (50, 70, 95, and 100%) for 30 min each, then pre-embedded with Epon: acetone 1:1 overnight and then included in pure Epon. The polymerization was carried out in an oven at 60°C for 48 h. Thin sections (80 nm) were cut with a Leica Ultracut R ultramicrotome, then stained with 1% uranyl acetate in methanol for 2 min and with Reynolds lead citrate for 5 min. The slices were observed with a Philips Tecnai 12 Biotwin microscope (Eindhoven, The Netherlands) at 80 kV.

### Statistical Analysis

Unless specified, all experiments were performed three times, with triplicates for each condition in each experimental run (the graphs show the average of 9–15 points). The results are expressed as the means ± standard errors. The data were analyzed by one-way or two-way analysis of variance (ANOVA), followed by Bonferroni’s *post hoc* test; *p* ≤ 0.05 was considered the threshold for statistical significance with 95% confidence interval (CI). If other statistical analysis was used, it was specified accordingly. Statistical analyses were performed using Prism software (GraphPad, United States). To test normality, we used the numerical method in SPSS Statistics software (IBM, United States) using the numerical method.

## Results

### *In vivo* Treatment With ANDRO Increases Brain Glucose Accumulation and Key Metabolic Markers

**FIGURE 1:**
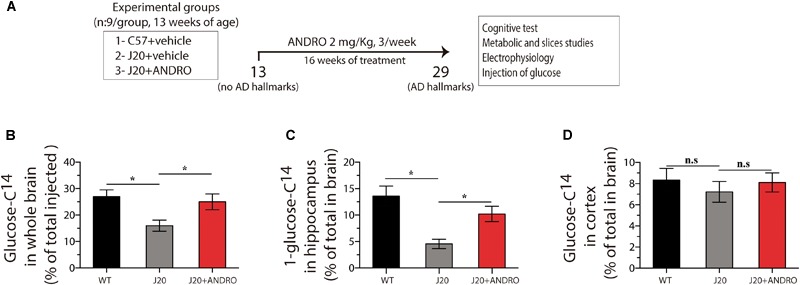
Treatment with ANDRO increases the in vivo uptake of glucose. Schematic of the protocol for assaying the effect of ANDRO administration in a presymtomatic stage **(A)**. After the indicated treatments, radioactive glucose was injected in the tail vein, and 15 min later, glucose uptake in the whole brain **(B)**, hippocampus **(C)**, and cortex **(D)** was measured. The magnitude of the decrease in glucose uptake was mitigated in J20 mice. ANDRO treatment improved glucose uptake in the J20 animals to levels similar to those of WT mice. Data represent the mean ± SEM of *n* = 6 (number of animals for treatment), *p* ≤ 0.05 (95% CI) was considered the threshold for statistical significance, Bonferroni test, *p*-values are indicated in the “Results” Section.

A reduction in cellular energy metabolism is an accurate marker of AD progression (Kapogiannis and Mattson, 2011; Cisternas and Inestrosa, 2017). Knowing that ANDRO increases glucose metabolism in symptomatic AD animals, we asked whether preventive administration of ANDRO was able to delay metabolic dysfunction. Mice were treated with ANDRO from postnatal week 13 for 16 weeks, and the treatment was finished when animals were 7 months old (Figure 1A). At this age, Tg animals show obvious cognitive deficits (Mucke et al., 2000; Saganich et al., 2006; Cheng et al., 2007; Tapia-Rojas and Inestrosa, 2018). First, we measured brain glucose accumulation by injecting radioactive glucose (D-[1-^14^C] glucose) via the tail vein. After 15 min, we measured the amount of radioactivity in the whole brain and in the regions most affected in AD, i.e., hippocampus and cortex. Glucose accumulation in the whole brain of J20 Tg mice was 38% lower than that observed in the WT mice. The ANDRO treatment increased the glucose levels of the J20 Tg whole brain near the WT values (one-way ANOVA, *p* < 0.05, followed by Bonferroni’s *post hoc* test, WT vs. J20 ^∗^*p* < 0.05; J20 vs. J20+ANDRO ^∗^*p* < 0.05) (Figure 1B). In the hippocampus, glucose accumulation was reduced ∼70% in J20 Tg mice relative to WT mice. The treatment of J20 Tg mice with ANDRO recovered glucose accumulation close to WT values (one-way ANOVA, *p* < 0.05, followed by Bonferroni’s *post hoc* test, WT vs. J20 ^∗^*p* < 0.05; J20 vs. J20+ANDRO ^∗^*p* < 0.05) (Figure 1C). No significant differences in cortical glucose accumulation were observed in the three animal groups of this study (one-way ANOVA, *p* < 0.05, followed by Bonferroni’s *post hoc* test, WT vs. J20 *p* = n.s.; J20 vs. J20+ANDRO *p* = n.s.) (Figure 1D). Hence, the accumulation of glucose is impaired in the hippocampus of J20 Tg mice, and it recovers to nearly normal values with presymptomatic ANDRO treatment.

**FIGURE 2:**
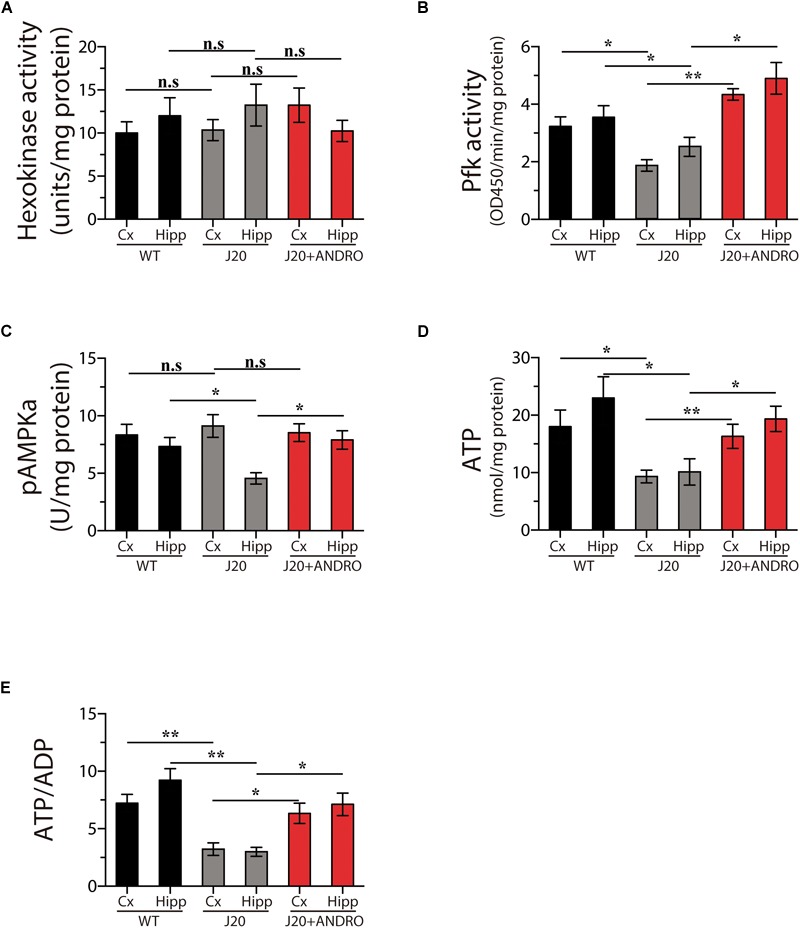
ANDRO treatment induces recovery of general brain metabolism. Treatment with ANDRO stimulated the enzymatic activity of two checkpoint proteins of glycolysis: HK **(A)** and Pfk **(B)**. Effects of treatments on the levels of AMPK, an important metabolic sensor of glucose metabolism **(C)**. ATP **(D)** and the ATP/ADP ratio **(E)** were measured to correlate the previous parameters with the energy status of cells. ANDRO restored the levels of both parameters in J20 mice. Data represent the mean ± SEM of *n* = 3 (independent experiments), each performed in triplicate. *p* ≤ 0.05 (95% CI) was considered the threshold for statistical significance, Bonferroni test, *p*-values are indicated in the “Results” Section.

We went a step further and examined the intracellular pathway of glucose utilization that is affected in the J20 Tg mice and targeted by ANDRO. After glucose is taken in by cells, it breaks down through glycolysis, a metabolic pathway that has several control points and includes the enzymes hexokinase (Hk) and phosphofructokinase (Pfk) and the brain metabolic sensor 5′ AMPK (Burkewitz et al., 2014). We measured the activity of Hk, Pfk, and AMPK and the activity of several metabolic markers in the hippocampus and cortex in all three groups in the present study. We did not find changes in Hk activity, the first control point for glucose utilization, in either the cortex or the hippocampus of WT, J20 Tg or J20 Tg+ANDRO mice (one-way ANOVA, *p* < 0.05, followed by Bonferroni’s *post hoc* test, Cx WT vs. Cx J20 *p* = n.s.; Cx J20 vs. Cx J20+ANDRO *p* = n.s.; Hipp WT vs. Hipp J20 *p* = n.s.; Hipp J20 vs. Hipp J20+ANDRO, *p* = n.s., Figure 2A). Interestingly, we observed a strong decrease in the activity of phosphofructokinase (Pfk) in both the hippocampus (2.56 ± 0.33) and the cortex (1.98 ± 0.21) of J20 Tg animals relative to control WT animals (3.88 ± 0.4 and 3.23 ± 0.34, respectively). The J20 Tg mice treated with ANDRO showed higher levels of Pfk activity in the cortex (4.35 ± 0.22) and hippocampus (4.97 ± 0.53), similar to the values observed in the WT mice (one-way ANOVA, *p* < 0.05, followed by Bonferroni’s *post hoc* test, Cx WT vs. Cx J20 ^∗^*p* = 0.05; Cx J20 vs. Cx J20+ANDRO ^∗∗^*p* = 0.01; Hipp WT vs. Hipp J20 ^∗^*p* = 0.05; Hipp J20 vs. Hipp J20+ANDRO, ^∗^*p* = 0.04, Figure 2B). As mentioned above, one of the most important metabolic regulators of glucose metabolism is the enzyme AMPK. Thus, we measured the activity of this enzyme in the cortex and hippocampus. We detected a decrease in the activity of this enzyme in the hippocampus of J20 Tg mice compared with WT mice (4.55 ± 0.49), but not in the cortex (9.11 ± 0.92), and activity values similar to those of WT animals were found in the J20 Tg+ANDRO mice (8.53 ± 0.49 and 7.93 ± 0.83, respectively) (one-way ANOVA, *p* < 0.05, followed by Bonferroni’s *post hoc* test, Cx WT vs. Cx J20 *p* = n.s.; Cx J20 vs. Cx J20+ANDRO *p* = n.s.; Hipp WT vs. Hipp J20 ^∗^*p* = 0.05; Hipp J20 vs. Hipp J20+ANDRO, ^∗^*p* = 0.05, Figure 2C). Taken together, the evidence shows that Pkf is the enzyme with the most severely affected activity in the cortex and hippocampus of J20 Tg mice and that ANDRO treatment is able to restore its activity to WT levels. Moreover, ANDRO mitigated the decrease in AMPK activity the hippocampus of J20 Tg mice.

We also measured the levels of ATP and the ATP/ADP ratio. We observed a decrease of ∼42% in the ATP levels in the cortex and hippocampus in the J20 mice, the treatment with ANDRO was able to recover in part the levels of ATP (one-way ANOVA, *p* < 0.05, followed by Bonferroni’s *post hoc* test, Cx WT vs. Cx J20 ^∗^*p* = 0.05; Cx J20 vs. Cx J20+ANDRO ^∗∗^*p* = 0.01; Hipp WT vs. Hipp J20 ^∗^*p* = 0.05; Hipp J20 vs. Hipp J20+ANDRO, ^∗^*p* = 0.05, Figure 2D). The recovery in the levels of ATP was directly related with the increase in the ratio ATP/ADP, a direct form to describe an increase in the synthesis of ATP, the ratio value of the J20+ANDRO was similar to the WT values (one-way ANOVA, *p* < 0.05, followed by Bonferroni’s *post hoc* test, Cx WT vs. Cx J20 ^∗∗^*p* = 0.01; Cx J20 vs. Cx J20+ANDRO ^∗^*p* = 0.05; Hipp WT vs. Hipp J20 ^∗∗^*p* = 0.01; Hipp J20 vs. Hipp J20+ANDRO, ^∗^*p* = 0.05, Figure 2E).

### Glucose Metabolism in Slices From J20 Tg Animals Is Altered and Rescued by ANDRO Treatment

**FIGURE 3:**
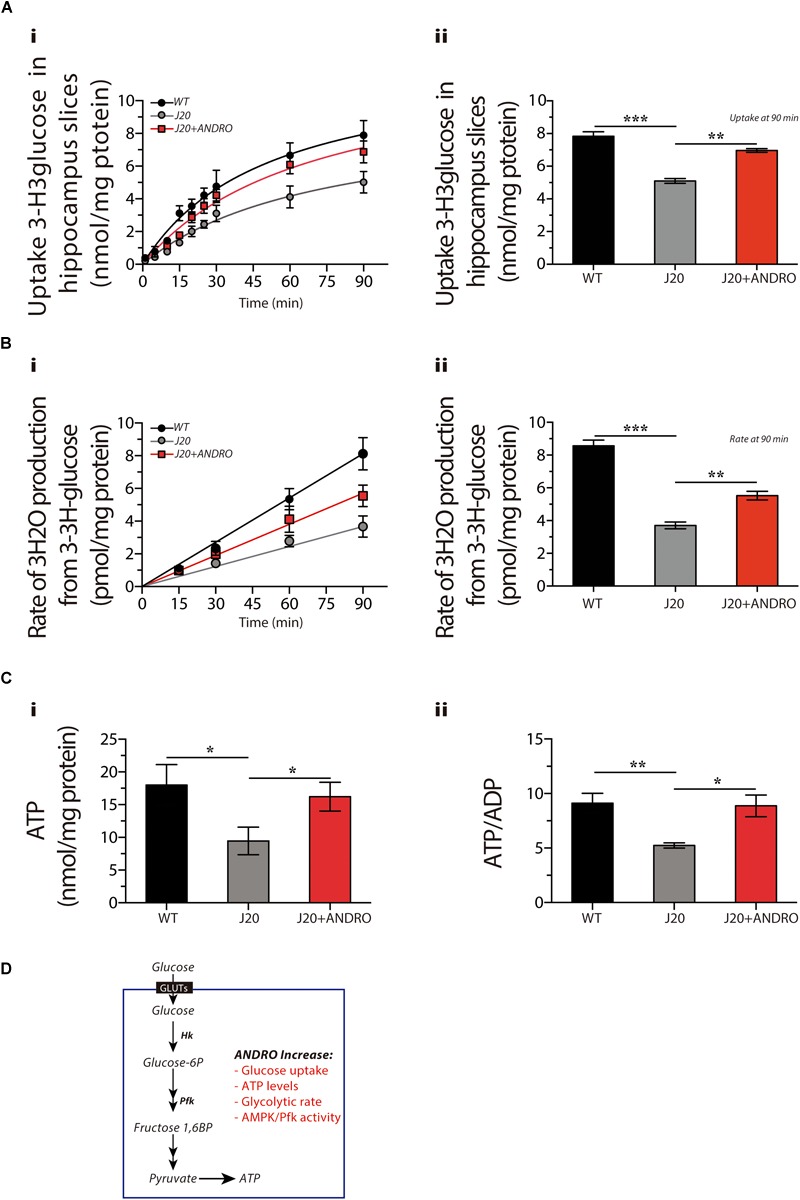
ANDRO promotes the utilization of glucose and enhances the production of ATP in hippocampal slices. The uptake of radioactive glucose **(Ai)** in slices obtained from WT, J20, and J20+ANDRO. Treatment with ANDRO increased the uptake of glucose in J20 slices to levels similar to WT at 90 min **(Aii)**. Glycolytic rate after treatment **(Bi)**. The decreased glycolytic rate of J20 slices was rescued by treatment with ANDRO **(Bii)**. Both ATP and the ATP/ADP ratio were decreased in slices from J20 mice, and both were increased in the J20+ANDRO mice (**Ci,ii**, respectively). **(D)** Presymptomatic treatment with ANDRO induces recovery of several metabolic markers, including glucose uptake, glucose utilization through glycolysis and ATP recovery. The data represent the mean ± SEM of *n* = 3 (independent experiments), each performed in triplicate. *p* ≤ 0.05 (95% CI) was considered the threshold for statistical significance, details in the “Results” Section.

Next, to evaluate whether the augmented metabolic rate observed in the hippocampus of J20 Tg mice treated with ANDRO correlates with glucose cellular uptake, we measured the uptake of radioactive glucose by hippocampal slices at different times (Figure 3Ai). Hippocampal slices were recovered from the three groups of animals. At 15 min, the uptake of glucose was 3.21 ± 0.16 nmol/mg protein, 1.32 ± 0.13 nmol/mg protein and 1.71 ± 0.09 nmol/mg protein for WT, J20 Tg and J20 Tg+ANDRO, respectively. Significantly less glucose uptake was observed at 90 min in J20 Tg animals (5.10 ± 0.15 nmol/mg protein) relative to the control WT (7.83 ± 0.27 nmol/mg protein). Interestingly, J20+ANDRO slices showed a glucose uptake of 6.97 ± 0.12 nmol/mg (For the three curves, two-way repeated measures ANOVA: interaction: *F*(18,60) = 14.46, *p* < 0.001; animal group factor: *F*(2,60) = 228.75; time: *F*(9,60) = 790.09; Figure 3A. For the last point (90 min), one-way ANOVA: *p* < 0.001, followed by Bonferroni *post hoc* test, WT vs. J20 ^∗∗∗^*p* < 0.001; J20 vs. J20+ANDRO ^∗∗^*p* < 0.01; Figure 3Aii), close to WT animals, suggesting that the reduced glucose uptake in J20 Tg mice can be restored by ANDRO treatment.

Another approach to measure the cellular utilization of glucose is to determine the glycolytic rate by measuring the radioactive ^3^H_2_O molecules generated from enolase activity (Figure 3Bi). A lower glycolytic rate was measured after 90 min in the J20 Tg mice (3.71 ± 0.20 pmol/mg protein) compared with the WT mice (8.57 ± 0.35 pmol/mg protein). Treatment with ANDRO partially rescued the level of activity after 90 min (5.52 ± 0.26 pmol/mg protein) [for the three curves, two-way repeated measures ANOVA: interaction: *F*(8,30) = 41.49, *p* < 0.001; animal group factor: *F*(2,30) = 123.31; time: *F*(4,30) = 622.49. For the last point (90 min), one-way ANOVA: *p* < 0.001, followed by Bonferroni *post hoc* test, WT vs. J20 ^∗∗∗^*p* < 0.001; J20 vs. J20+ANDRO ^∗^*p* < 0.05; Figure 3Bii]. Finally, to correlate the uptake of glucose and the glycolytic rate with ATP production, we measured ATP levels and ATP/ADP ratio (Figure 3Ci,ii, respectively). The brain slices obtained from J20 Tg animals showed a decrease of 50% in the levels of ATP and a similar decrease in the ATP/ADP ratio (5.23 ± 0.24) compared to what was observed in the WT mice (9.12 ± 0.93). The slices obtained from the J20 Tg+ANDRO group showed higher levels of ATP (one-way ANOVA, *p* < 0.05, followed by Bonferroni’s *post hoc* test, WT vs. J20 ^∗^*p* = 0.05; J20 vs. J20+ANDRO ^∗^*p* = 0.05, Figure 3Ci) and ATP/ADP ratios (8.87 ± 0.99), similar to WT mice (one-way ANOVA, *p* < 0.05, followed by Bonferroni’s *post hoc* test, WT vs. J20 ^∗∗^*p* = 0.01; J20 vs. J20+ANDRO ^∗^*p* = 0.05, Figure 3Cii). Our results indicate that preventive treatment with ANDRO was able to rescue several markers of energy metabolism (Figure 3D) that were deficient in the J20 Tg mice. To study whether these findings correlate with an improvement in neuronal activity, we analyzed and compared the cognitive performance of the Tg animals and the effect of ANDRO on neuronal plasticity.

### Early Administration of ANDRO Rescues Cognitive Deterioration

**FIGURE 4:**
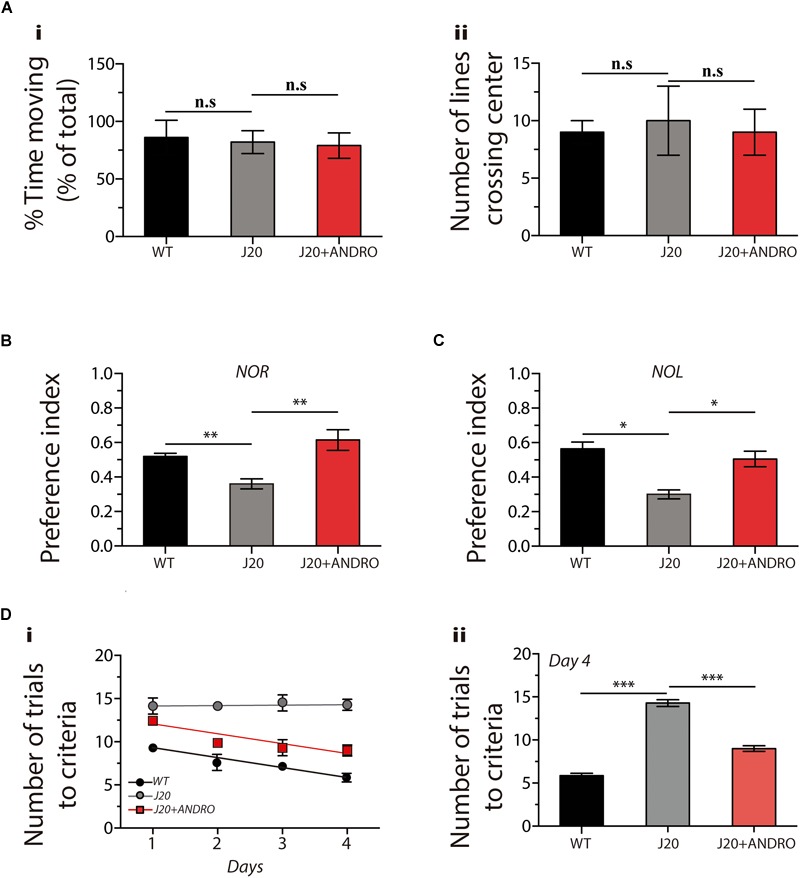
The administration of ANDRO improves cognitive performance in a transgenic model of AD. After the indicates treatments, cognitve test related to hippocampal function were performed: large open field **(A)**, NOR **(B)**, NOL **(C)** and memory flexibility **(D)**. Data represent the mean ± SEM of *n* = 9 (number of animals), *p* ≤ 0.05 (95% CI) was considered the threshold for statistical significance, details in the “Results” Section.

We explored whether administration of ANDRO in the presymptomatic stage delays the cognitive impairment of J20 Tg mice. First, we first studied the activity of the mice using the open-field test. This test measures the time spent moving and the number of times that the animal crosses the center of the cage. Both parameters are used to rule out movement or stress problems. We observed no significant changes among groups, showing that the basal status does not affect the animal’s performance (one-way ANOVA, *p* < 0.05, followed by Bonferroni’s *post hoc* test, WT vs. J20 *p* = n.s.; J20 vs. J20+ANDRO *p* = n.s., Figure 4Ai,ii).

To evaluate memory, we performed NOR and NOL tests, both associated with hippocampal function. In the NOR test, we observed a significant decrease in the preference index in the J20 Tg mice treated with vehicle in comparison with the WT mice. However, in the J20 Tg animals treated with ANDRO, we observed preference indexes similar to those of WT mice (one-way ANOVA, *p* < 0.05, followed by Bonferroni’s *post hoc* test, WT vs. J20 ^∗∗^*p* = 0.01; J20 vs. J20+ANDRO ^∗∗^*p* = 0.01, Figure 4B). In the NOL test, we observed that the treatment with ANDRO was able to recover the cognitive decline observed in the J20 Tg mice treated with vehicle (one-way ANOVA, *p* < 0.05, followed by Bonferroni’s *post hoc* test, WT vs. J20 ^∗^*p* = 0.05; J20 vs. J20+ANDRO ^∗^*p* = 0.05, Figure 4C). The results suggest that ANDRO protects against memory loss in J20 Tg mice.

Next, we performed a highly sensitive memory flexibility test to study learning and memory performance (Chen et al., 2000). On the first trial day, the WT mice needed eight trials to reach criterion, while the J20 Tg mice treated with ANDRO or vehicle needed 13 and 14 trials, respectively (Figure 4D). However, after 4 days of testing, the WT mice needed six trials to reach criterion, whereas the J20 Tg mice needed the same 14 trials, showing a decrease in learning/memory performance (Figure 4Di). Interestingly, after 4 days, the J20 Tg+ANDRO mice needed only nine trials to reach criterion, showing that the treatment with ANDRO was able to delay the cognitive decline that affected memory/learning [Figure 4D: two-way repeated measures ANOVA: interaction *F*(6,72) = 4.26, *p* < 0.01; animal group factor: *F*(2,72) = 222.54; days: *F*(3,72) = 12.65. Figure 4Dii: we compared among groups the performance at the fourth day, observing significant differences in all groups. Figure 4Dii: one-way ANOVA, *p* < 0.001, followed by Bonferroni *post hoc* test, WT vs. J20 ^∗∗∗^*p* < 0.001; J20 vs. J20+Andro ^∗∗∗^*p* < 0.001].

### Deficiencies in Basal Activity and Synaptic Plasticity Occurs in J20 Animals and Are Rescued by ANDRO

**FIGURE 5:**
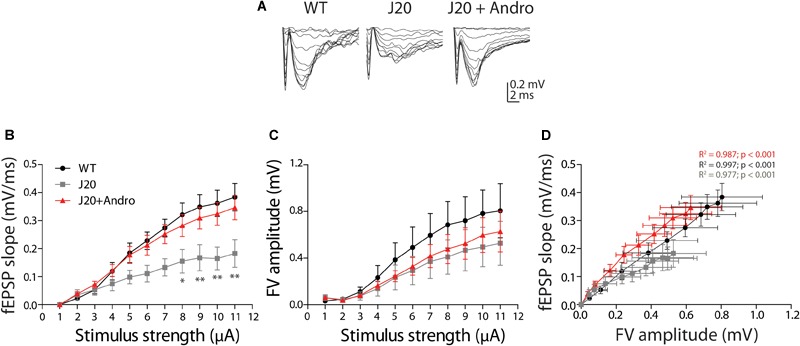
Input–output curves showing reduced basal synaptic transmission in J20 Tg mice. Representative fEPSPs **(A)** evoked in response to successive increments of current intensity (from 1 to 11 μA), in WT (black dots), J20 (gray squares) and J20+ANDRO (red triangles). Input–output curves of fEPSP slope **(B)** and FV amplitude **(C)**, both against stimulus intensity, demonstrating deficient synaptic transmission. Linear regression plot of fEPSP slopes dependence on FV amplitude **(D)**, evidenced that Tg animals cannot sustain responses with high stimulation. In B, WT vs. J20+ANDRO are significantly different from J20 [two-way repeated measures ANOVA: interaction: *F*(20,220) = 1.63, *p* > 0.05; animal group factor: *F*(2,220) = 29.35, *p* < 0.001; stimulation intensity: *F*(10,220) = 30.61, *p* < 0.001, followed by Bonferroni *post hoc* test: WT vs. J20 at 8 μA ^∗^*p* < 0.05, 9 to 11 μA ^∗∗^*p* < 0.01; J20 vs. J20+Andro at 8 μA ^∗^*p* < 0.05, 9 to 11 μA ^∗∗^*p* < 0.01; WT vs. J20+Andro *p* = n.s.]. In panel **(D)**, the data were fitted with a linear regression: *R*^2^ = 0.99, 0.98, and 0.99 correspondingly; and slopes for WT = 0.459 ± 0.008; J20 = 0.3125 ± 0.016; J20+ANDRO = 0.530 ± 0.021. ANCOVA analysis between WT and J20: *F*(1,19) = 12.51, *p* = 0.002; WT and J2+ANDRO: *F*(1,19) = 49.87, *p* < 0.001; and J20 and J20+ANDRO = *F*(1,19) = 37.64, *p* < 0.001, gave significant differences among all linear regressions.

To determine whether cognitive disabilities and the restoration caused by ANDRO have a synaptic correlate, we studied the electrophysiological parameters involved in synaptic transmission and plasticity mechanisms. To do this, we studied synaptic transmission in the well-known synapse formed by Schaffer collaterals in the CA1 hippocampal region in 7-month-old WT, J20 and J20+ANDRO mice. We stimulated the collateral axons and recorded them at the level of the CA1. We applied increasing levels of current intensities that evoked increased fEPSP slopes and FV amplitudes, and we constructed an input–output relationship (Figure 5A). As shown, the plot of fEPSP slope against stimuli amplitude demonstrated that J20 animals have a deficiency in basal synaptic transmission compared to WT animals or to Tg animals treated with ANDRO [Figure 5B, measured at the maximum current intensity: WT = 0.38 ± 0.05 mV/ms; J20 = 0.18 ± 0.05 mV/ms; J20+ANDRO = 0.35 ± 0.04 mV/ms; two-way repeated measures ANOVA: interaction: *F*(20,220) = 1.63, *p* > 0.05; animal group factor: *F*(2,220) = 29.35, *p* < 0.001; stimulation intensity: *F*(10,220) = 30.61, *p* < 0.001, followed by Bonferroni *post hoc* test, ^∗^*p* < 0.05, ^∗∗^*p* < 0.01]. The input–output relationship based on the FV versus stimulus amplitude did not show a difference between animals, demonstrating that the number of axons recruited and their excitability did not differ between animal groups (measured at the maximum current intensity: WT = 0.8 ± 0.23 mV/ms; J20 = 0.53 ± 0.19 mV/ms; J20+ANDRO = 0.63 ± 0.17 mV/ms) (Figure 5C). We also plotted the correlation between the FV amplitude and the fEPSP slope (Figure 5D). Interestingly, the regression analysis resulted in a significant positive association between fEPSP slopes values and fiber volley amplitude for our three treatments [WT_slope_ = 0.459 ± 0.008, *R*^2^ 0.99, *F*(1,9) = 3498.13, *p* < 0.001; J20_slope_ = 0.3125 ± 0.016, *R*^2^ 0.98, *F*(1,9) = 390.18, *p* < 0.001; J20+ANDRO_slope_ = 0.530 ± 0.021, R^2^ 0.99, *F*(1,9) = 659.18, *p* < 0.001; Figure 5D], demonstrating that the strength of synaptic transmission per activated axon is different among groups. Thus, J20 Tg mice that show a relatively small slope correlation also show an increase when treated with ANDRO. These data suggest that is not the number of axons but a synaptic mechanism that is altered in the J20 mice and that ANDRO pretreatment is able to revert.

**FIGURE 6:**
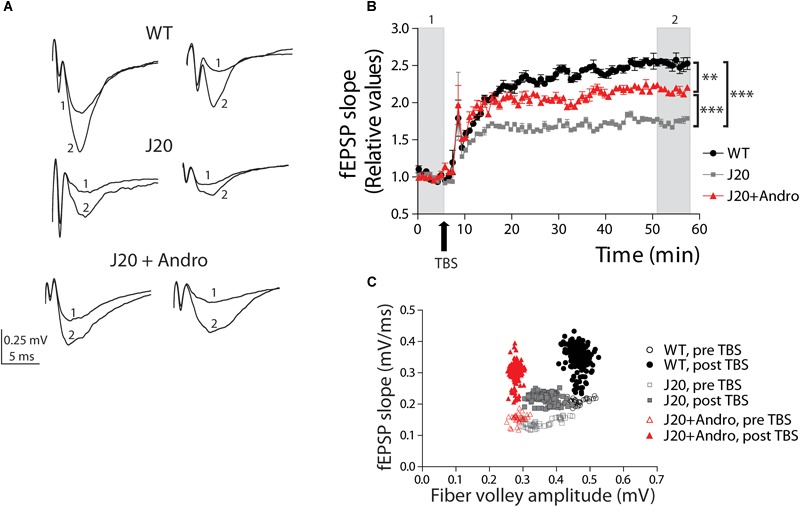
Pretreatment with ANDRO increases the level of LTP. Two representative traces of field EPSPs **(A)** recorded before (1) and after LTP induction (2) in each animal group. WT (black dots), J20 (gray squares), and J20+ANDRO (red triangles). Twenty minutes of basal activity was recorded **(B)** before LTP was induced by theta burst stimulation (TBS, arrow), as explained in the “Results” Section. The fEPSPs slope measurements after LTP induction were normalized to the slope measurements during basal time. Treatment with ANDRO increased the amount of LTP compared to J20, but to a lesser extent than WT. After TBS, comparing the averages of the last 10 min among groups, all three curves become significantly different (one-way ANOVA, *p* < 0.001, followed by Bonferroni *post hoc* test WT vs. J20, ^∗∗∗^*p* < 0.001; WT vs. J20+ANDRO, ^∗∗^*p* < 0.01; J20 vs. J20+ANDRO, ^∗∗∗^*p* < 0.001). **(C)** Correlation plot between FV amplitude and fEPSP slope before and after LTP induction. WT: pre-TBS (open black circles), post-TBS (solid black circles); J20: pre-TBS (open gray squares), post-TBS (solid gray squares); J20+ANDRO: pre-TBS (open red triangles), post-TBS (solid red triangles). The correlation between FV amplitude and fEPSP slope was found using Pearson’s correlation. Using Fisher ‘*r*’ to ‘*z*’ transformation, we used *z*-scores to compute the significance of the difference when we compare two correlation coefficients before TBS (open symbols). The same analysis was performed to compare correlation coefficients after TBS (closed symbols). After LTP induction, we found significant differences in all group comparisons (for details, see main text). Data represents the mean ± SEM from 16 slices of 8 (WT) mice; 18 slices of 6 (J20) mice; 18 slices of 6 (J20+Andro) mice. Further details, see Section “Materials and Methods.”

We next investigated hippocampal synaptic plasticity in the form of LTP. Previously, our laboratory demonstrated that J20 Tg has several characteristics of the disease (Tapia-Rojas and Inestrosa, 2018). Then, we hypothesized that some of these defects would have consequences in the mechanisms that generate long-term synaptic plasticity. After obtaining a stable baseline of evoked fEPSPs, we applied TBS to induce LTP. Two examples of fEPSPs evoked before and after TBS in WT, J20, and J20+ANDRO, are shown (Figure 6A). The analysis of the fEPSP slope after TBS relative to the slope before TBS was plotted against time and is shown in the graph (Figure 6B). The shape of LTP induction shows a slow time course until reaching a steady state in all three groups. This slow induction might be related to the differential equilibrium between long-term potentiation and depression (LTD), which shifts toward LTD in aged animals (Megill et al., 2015), or to age-dependent differences in the induction of LTP. Despite the differences in LTP amplitude observed in the plot, WT, J20 and J20+ANDRO showed significant potentiation above the basal amplitude (Figure 6B). The averaged fEPSP slope value of J20 Tg mice (60 ± 5% of baseline) after an hour of LTP induction is smaller than WT (150 ± 10% of baseline), while J20+ANDRO (110 ± 3% of baseline) shows better potentiation than J20 Tg (one-way ANOVA, *p* < 0.001, followed by Bonferroni *post hoc* test, WT vs. J20 ^∗∗∗^*p* < 0.001; J20 vs. J20+ANDRO ^∗∗∗^*p* < 0.001; WT vs. J20+ANDRO ^∗∗^*p* < 0.01). This means that treatment with ANDRO increases the potentiation level in the transgenic mice almost twofold. The differences between animal groups under an LTP protocol should be attributable to postsynaptic mechanisms. To demonstrate that in our experiments, we first measured the range of FV amplitudes before and after LTP induction (WT before: 0.47 ± 0.006 mV, after: 0.46 ± 0.001 mV, *p* = ns; J20 before: 0.41 ± 0.005 mV, after: 0.39 ± 0.002, *p* = ns; J20+ANDRO before: 0.29 ± 0.003 mV, after: 0.28 ± 0.001 mV, *p* = ns, by two-way ANOVA and Bonferroni *post hoc* test). Then, we plotted the input–output function of these experiments. We averaged the FV amplitudes of all experiments and plotted them against the averaged fEPSP slope of all experiments, both during basal stimulation (open symbols) and after LTP induction (solid symbols) (Figure 6C). Therefore, every point represents the averaged FV value and the averaged slope of the evoked response. We analyzed the correlation between these two variables to quantify the degree to which they are related, and whether they differ among the three groups. By Pearson’s correlation we found the correlation coefficient ‘*r*’ of each group during pre-TBS condition (open symbols) (by Pearson’s correlation WT: *r* = 0.5290, J20: *r* = 0.0152, J20+ANDRO: *r* = 0.3244). Using Fisher ‘*r*’ to ‘*z*’ transformation, we used *z*-scores to compute the significance of the difference when we compare two correlation coefficients (WT vs. J20: z = 2.11, two-tailed *p* = 0.032; J20 vs. J20+ANDRO: *z* = 0.93, two-tailed *p* = 0.352; WT vs. J20+ANDRO: *z* = -1.18, two-tailed *p* = 0.238). When *z* is positive means that the ‘*r*’ of that group (e.g., WT) is greater than the one which is compared to (e.g., J20); if *z* its negative, the ‘*r*’ of the first group (e.g., J20) is smaller than the one which is compared to (e.g., J20+ANDRO). The first case is the only one significant: WT correlation coefficient is greater than the ‘*r*’ of J20. Then, we found the correlation coefficient ‘*r*’ of each group during post-TBS condition (closed symbols) (by Pearson’s correlation WT: *r* = -0.1430, J20: *r* = 0.3657, J20+ANDRO: *r* = 0.07478). As above, *z*-scores were computed to assess the significance of the difference between two correlation coefficients (WT vs. J20: *z* = -5.78, two-tailed *p* = 0; J20 vs. J20+ANDRO: *z* = 3.39, two-tailed *p* = 0.0007; WT vs. J20+ANDRO: *z* = -2.4, two-tailed *p* = 0.016). We found significant differences in all comparisons: WT and J20+ANDRO are significantly less correlated than J20, what is explained by their increment in slope after TBS. Interestingly, the response to ANDRO pretreatment shows that the system appears to be optimized to generate a broad range of responses or synaptic transmission with reduced axonal recruitment activity. Assuming that the number of axons is consistent among the different animal groups (Figure 5C), we propose that ANDRO acts at the presynaptic region and modulates the postsynaptic machinery to optimize the strength of the response.

**FIGURE 7:**
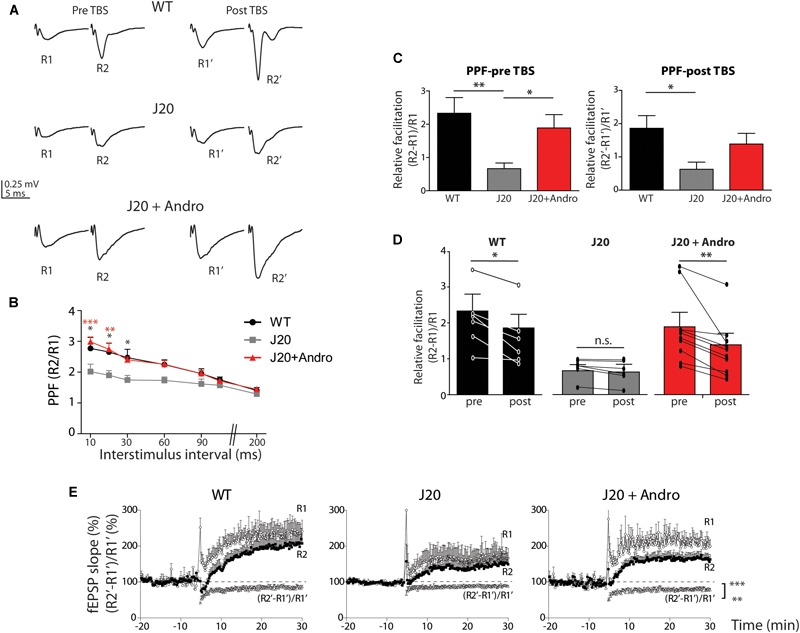
Short-term plasticity is rescued by pretreatment with ANDRO. Representative traces **(A)** of two evoked fEPSPs separated by 50 ms before (pre-TBS) and after LTP induction (post-TBS) are shown. The graph **(B)** shows the ratio of the second fEPSP slope to the first fEPSP slope (R2/R1), recorded at different interval times: 10, 20, 30, 50, 60, 80, 100, 150, and 200 ms. WT (black dots), J20 (gray squares) and J20+ANDRO (red triangles). The values are expressed as mean ± SEM [two-way repeated measures ANOVA: interaction: *F*(12,105) = 1.09, *p* > 0.05; animal group factor: *F*(2,105) = 19.87, *p* < 0.001; ISI: *F*(6,105) = 18.58, *p* < 0.001, followed by Bonferroni *post hoc* test: WT vs. J20 at 10, 20 and 30 ms ^∗^*p* < 0.05, beyond *p* = n.s.; J20 vs. J20+Andro at 10 ms ^∗∗∗^*p* < 0.001, at 20 ms ^∗∗^*p* < 0.01, beyond *p* = n.s.; WT vs. J20+Andro *p* = n.s.]. For the same stimulus point, the three curves were compared **(C)** show the relative facilitation during the baseline period [(R2 – R1)/R1], pre-TBS, left plot (WT: 2.3 ± 0.5; J20: 0.7 ± 0.2; J20+ANDRO: 1.9 ± 0.4; one-way ANOVA, *p* < 0.05, followed by Bonferroni *post hoc* test, ^∗^*p* < 0.05, ^∗∗^*p* < 0.01) and after LTP induction [(R2’ – R1’)/R1’], post-TBS, right plot (WT: 1.9 ± 0.4; J20+ANDRO: 1.4 ± 0.3; J20: 0.6 ± 0.2, one-way ANOVA, *p* < 0.05, followed by Bonferroni *post hoc* test, ^∗^*p* < 0.05). The average of each bar was calculated over all the points during the baseline period and the last 20 min recorded during LTP. Also shown **(D)** are the same data as in **(C)**, but plotted by animal group. Comparing facilitation within each group per separated, we showed that facilitation pre-TBS is significantly different than facilitation post-TBS in WT [two-way repeated measures ANOVA: interaction: *F*(2,14) = 2.68, *p* > 0.05; pre and post TBS condition: *F*(1,14) = 18.33, *p* < 0.001; animal group factor: *F*(2,14) = 8.43, *p* < 0.01, followed by Bonferroni *post hoc* test, WT pre vs. post TBS ^∗^*p* < 0.05, J20 pre vs. post TBS *p* > 0.05, J20+Andro pre vs. post ^∗∗^*p* < 0.01]. Plot of R1’ and R2’ (%) and the percentage of attenuation [(R2’ – R1’)/R1’] **(E)**, revealing a reduced degree of response attenuation in J20 during the first 30 min after LTP induction (one-way ANOVA, *p* < 0.001 followed by Bonferroni *post hoc* test, WT vs. J20 ^∗∗∗^*p* < 0.001, J20 vs. J20+Andro ^∗∗∗^*p* < 0.001, WT vs. J20+Andro ^∗∗^*p* < 0.01). Data represents the mean ± SEM from 16 slices of 8 (WT) mice; 18 slices of 6 (J20) mice; 18 slices of 6 (J20+Andro) mice.

To decipher the mechanisms targeted by ANDRO, we analyzed the PPF, which is a presynaptic form of plasticity. This protocol measures the probability of release of presynaptic terminals. We evoked two presynaptic spikes separated at different times (10–200 ms) and recorded the postsynaptic responses (fEPSP) as R1 and R2. The resultant PPF index is shown in Figure 7B. The three groups showed facilitation (ratio above 1) at a range of interstimulus intervals (10–90 ms). At this time, WT is undistinguishable from J20 Tg+ANDRO, and J20 shows a significantly lower ratio level [WT: 2.77 ± 0.36; J20: 2.02 ± 0.24; J20+ANDRO: 2.98 ± 0.16; two-way repeated measures ANOVA: interaction: *F*(12,105) = 1.09, *p* > 0.05; animal group factor: *F*(2,105) = 19.87, *p* < 0.001; interstimulus intervals: *F*(6,105) = 18.58, *p* < 0.001, followed by Bonferroni *post hoc* test, ^∗^*p* < 0.05, ^∗∗^*p* < 0.01, ^∗∗∗^*p* < 0.001]. We also measured the PPF between pulses before and after TBS (Figure 7A,C,D). The application of two pulses (R1 and R2) separated by 50 ms during the experiment allowed us to determine the degree of facilitation and how this value changed with the induction of LTP. Figure 7A shows two examples of WT, J20 and J20+ANDRO, with the two pre-TBS evoked responses (R1 and R2) and its corresponding (R1’ and R2’) 40 min after application of TBS. We plotted the relative facilitation [(R2 - R1)/R1] before and after TBS [(R2’ - R1’)/R1’]. We showed that even during basal transmission, the facilitation index differs significantly between WT and J20 and between J20 and J20+ANDRO (WT: 2.3 ± 0.5; J20: 0.7 ± 0.2; J20+ANDRO: 1.9 ± 0.4, by one-way ANOVA and *post hoc* Bonferroni test, ^∗∗^*p* < 0.01, ^∗^*p* < 0.05) (Figure 7C). This finding confirms that the facilitation index determined by PPF is severely defective in J20, while ANDRO is able to revert this condition, showing values close to WT (Figure 7C). Strikingly, the facilitation index after TBS shows that facilitation decreases in WT and J20+ANDRO, which is expected if the first pulse increases more in relation to the increment in the second pulse (WT: 1.9 ± 0.4; J20+ANDRO: 1.4 ± 0.3; J20: 0.6 ± 0.2, by one-way ANOVA and *post hoc* Bonferroni test, ^∗^*p* < 0.05). A summary of both sets of graph data, showing the comparison within groups, is shown below (Figure 7D). These data indicate another characteristic of the plasticity process, that is, the dynamics of PPF during LTP. TBS induces LTP but also attenuation of PPF responses. This suggests that the expression of LTP can induce changes in the presynaptic component (Schulz et al., 1994). The plot of R1 and R2 shows the dynamic change in synaptic transmission after TBS (WT: R1 = 232 ± 29%, R2 = 210 ± 27%; J20: R1 = 166 ± 31%, R2 = 153 ± 27%; J20+ANDRO: R1 = 217 ± 23%, R2 = 163 ± 15% all relative to baseline, *p* < 0.01) and on the PPF, showing attenuation (mean value relative to baseline: WT = 82 ± 0.4%, J20 = 92 ± 0.24%, J20+ANDRO = 77 ± 0.3%, by one-way ANOVA and *post hoc* Bonferroni test, ^∗∗∗^*p* < 0.001, ^∗∗^*p* < 0.01) (Figure 7E). This previously reported phenomenon (Schulz et al., 1994; Wang and Kelly, 1997), suggests that high frequency activity causes plasticity changes both at the level of LTP and PPF. Although we have not explored the mechanism associated with this effect, whether presynaptic or postsynaptic, it is interesting that the amount of attenuation is lesser in J20 and greater with ANDRO.

### ANDRO Treatment Prevents Increases in the Size of Hippocampal Synapses

**FIGURE 8:**
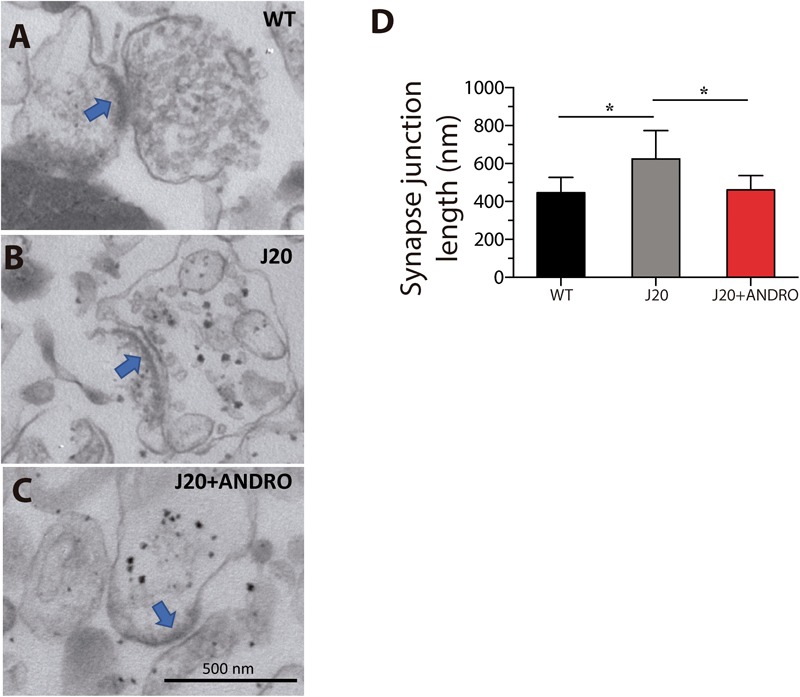
Effect of ANDRO on synapse length. Electron micrographs of synaptosomes from wild-type **(A)**, J20 Tg **(B)** and J20 Tg+ANDRO mice **(C)**. The graph **(D)** shows a significant increase in the synapse length in the J20 Tg mice in comparison with WT mice and J20 Tg+ANDRO. Images are representative of three different experiments. The data represent the mean ± SEM of *n* = 3 (independent experiments, one-way ANOVA and post Bonferroni Multiple Comparison Test was performed, ^∗^*p* < 0.05 and 95% CI).

It has been reported from postmortem AD brains that the number of synapses in the hippocampus is dramatically decreased, but the remaining synapses are large in sides, which led to the idea that there are compensatory mechanisms working to overcome the synapse loss (Dekosky and Scheff, 1990; Scheff et al., 1990). Here, we biochemically prepared synaptosome-enriched fractions from the hippocampi of the three groups of animals in the study. Figure 8 shows electron micrographs of synaptic junctions of WT, J20 Tg and J20 Tg+ANDRO mice. The right panel shows a quantitative analysis of the synaptic junction length. J20 Tg showed a larger synaptic junction (624 ± 149 nm) in comparison with the control (469 ± 52 nm). ANDRO treatment brought the length of the synaptic junction for the J20 Tg close to the control value (462 ± 75 nm) (one-way ANOVA, *p* < 0.05, followed by Bonferroni’s *post hoc* test, WT vs. J20 *p* = 0.05; J20 vs. J20+ANDRO *p* = 0.05, Figure 8).

## Discussion

Although first described over 100 years ago, the etiology of AD is not well understood, which limits the ability to treat the disease or slow its progression (Querfurth and LaFerla, 2010; Serrano-Pozo et al., 2011). AD is the most common form of dementia, affecting approximately 10% of individuals over the age of 65 and approximately 50% of people 85 years and older (Association, 2013). Most cases are sporadic, yet approximately 1–2% are genetically linked and can be distinguished by early-onset dementia (Bettens et al., 2013). Increasing longevity in humans, combined with the high incidence of AD in older adults, will only exacerbate the global public health cost (Association, 2013; Chen and Zhong, 2013). In this context, the prevention of AD has emerged as a real strategy against the AD burden. However, no correlation has been described between presymptomatic modulation of any specific signaling pathway and AD marker expression in AD mouse models, including aspects related to metabolic dysfunction and impairment of synaptic function. In the present work, we studied whether the preventive administration of ANDRO, a well-described agonist of canonical Wnt signaling, is able to delay the specified markers of AD.

During the onset and progression of AD, a decrease in the utilization of glucose by brain cells has been described, leading to low ATP levels, which could be a critical step that partially explains the beginning of the synaptic failure described in AD patients and in AD animal models. The brain is characterized by a high rate of glucose consumption, and defective glucose utilization has been described in various diseases related to dementia, including AD (Chen and Zhong, 2013; Cisternas and Inestrosa, 2017). The dysregulation of glucose metabolism in AD has gained importance as a therapeutic target because the administration of hormones that stimulate glucose metabolism, such as insulin and glucagon-like peptide 1 (GLP-1), improves cognitive responses in humans diagnosed with AD, as well as in mouse models of AD (Craft et al., 2012; Chapman et al., 2013; Freiherr et al., 2013), suggesting that the dysregulation of glucose in the brain could be critical in the onset and progression of AD as well as the progression of other metabolic diseases including diabetes. However, the effects of the hormones insulin and GLP-1 on downstream pathways are not well understood and represent an interesting field of research; in fact, several reports implicate Wnt signaling in the regulation of glucose metabolism (Cisternas and Inestrosa, 2017).

Several pathways have been postulated as molecular links between glucose metabolism and brain function, including Wnt signaling. Dysregulation of canonical Wnt signaling through a loss or gain of function has been linked to the progression of various diseases, including diabetes mellitus type II and AD (Inestrosa and Toledo, 2008; Farias et al., 2010; Toledo and Inestrosa, 2010; Inestrosa et al., 2012; Oliva et al., 2013; Cisternas et al., 2014b; Inestrosa and Varela-Nallar, 2014; Rios et al., 2014). The involvement of the Wnt pathway in the regulation of glucose metabolism has regained prominence in recent years, as studies in humans have suggested different components of the Wnt pathway as risk factors for the development of diseases such as DMTII and age-related dementia. However, the final effect depends on whether canonical or non-canonical Wnt signaling is affected (Lyssenko, 2008; Schinner, 2009; Shao et al., 2013). Despite all these efforts, the relationship between this signaling pathway and glucose metabolism in the brain has not been clearly established (Inestrosa and Arenas, 2010; Inestrosa and Varela-Nallar, 2014; Rios et al., 2014).

Recently, we have shown that acute treatment with the ligand Wnt3a induces a large increase in glucose uptake in neurons without changing the expression or localization of GLUT3, a glucose transporter expressed mostly in neurons (Cisternas et al., 2016a). We also observed that Wnt3a treatment increased the activation of the metabolic sensor Akt. Moreover, the increases in HK activity and glycolytic rates depended on activation of the Akt pathway. Furthermore, we did not observe changes in the activity of G6PDH or in the PPP. The effect of Wnt3a did not depend on the transcription of Wnt target genes or on synaptic changes (Cisternas et al., 2016a). Moreover, a recent study showed that the activation of canonical Wnt signaling by the *in vivo* administration of Wnt agonists, such as ANDRO and lithium, was able to induce a significant metabolic improvement and rescue cognitive performance in a transgenic model of advanced AD (Cisternas et al., 2018). Therefore, Wnt signaling stimulates the use of glucose in cortical neurons through glycolysis to satisfy the high energy demand of these cells, a critical effect that would explain the improvements in cognitive performance and synaptic plasticity observed in the presence of Wnt ligands. However, the final effect on neuronal metabolism depends on the specific Wnt pathway activated.

In AD, one of the most severely affected brain regions is the hippocampus, a region critical in the generation of new memories and other cognitive functions (Eichenbaum, 2014, 2017). The neural network of the hippocampus implies a high level of neuronal activity that is accompanied by a high energy demand; in fact, in WT animals, a significant proportion of the injected glucose was detected in the hippocampus. Interestingly, in the presymptomatic J20 mice, a significant decrease in the accumulation of glucose was observed in the hippocampus; therefore, although the animals presented normal cognitive performance, an early decrease in the utilization of glucose was detected. The latter supports the idea that metabolic dysfunction is one of the first molecular alterations in AD (Kapogiannis and Mattson, 2011). Given that both ATP and glucose uptake decreased in J20 mice, our data suggest that the metabolic dysfunction occurred mainly in neurons, since it is known that these cells use glucose mainly through glycolysis to generate ATP. The recovery in both metabolic parameters could be explained by a dual effect of ANDRO. First, ANDRO could modulate the activity of key metabolic enzymes such as Pfk, and consequently the increase in the activity of this enzyme could trigger an increase in the generation of pyruvate which is the main substrate for the mitochondrial machinery to produce ATP. But we cannot rule out a direct effect on the activity of the mitochondria, what would be subject for another study. Another action of ANDRO could be the activation of some metabolic sensors, such as AMPK, promoting cells to initiate an adaptive response to recover the general metabolic state (Ríos et al., 2018).

The improvement in the utilization of glucose and the generation of ATP both *in vivo* and in hippocampal slices was correlated with significant recovery of learning and memory function to levels similar to those of WT mice. Since this observation suggests a synaptic effect of ANDRO, we used electrophysiological approaches to explore synaptic function in J20 Tg mice with and without ANDRO treatment. We found severe deficiencies in synaptic transmission in the J20 Tg mice. The input–output curve revealed a deficiency in the level of response evoked by a given stimulus strength. However, it is unlikely that the mutant mice had fewer available axons or less excitable axons than the WT mice, since the FV plot did not show any difference among groups. The FV component and the evoked fEPSP showed a linear correlation in all groups. However, the same stimulation intensity activates fewer axons in J20 Tg mice than in the other two groups. These results suggest that the intrinsic activation of the axon and not the number of axons is altered in J20 Tg mice. On the other hand, the number of release sites or the efficiency of neurotransmitter release could be affected in the J20 Tg mice. Interestingly, treatment with ANDRO prevents the phenotypic effect of the transgene. These observations could, to some extent, be explained by observations made in humans wherein postmortem brains of AD patients have a significant decrease in the number of synapses and an increase in synaptic size (Dekosky and Scheff, 1990; Scheff et al., 1990), suggesting that a compensatory mechanism might strengthen the remaining synapses. Here, we explore one feature of the synapses in a biochemical preparation of synaptosomes. We found an increase in the length of the synaptic junction in the J20 Tg mice, mimicking the observation in human patients. Preventive treatment with ANDRO avoided that phenotype. Overall, this early cognitive and metabolic protection are the first such effect reported for the diterpene ANDRO since a previous report showed the ability of ANDRO to decrease neuropathology in AD mice; however, in that study, ANDRO treatment of AD mice started at 7 and 12 months of age, when the cognitive deficits of the disease were already evident (Serrano et al., 2014; Tapia-Rojas and Inestrosa, 2018).

We also studied classical long-term plasticity in the form of LTP. We found that all three groups of animals showed LTP, with J20 Tg mice showing the lowest LTP. Treatment with ANDRO significantly increased the LTP level in J20 Tg mice, which reached an intermediate level between those of the mutant and the WT mice. These data can explain, to some extent, the observation in the memory flexibility test, where ANDRO partially reestablished the normal phenotype. A very striking observation is that ANDRO rescues the deficit in synaptic transmission. In other words, the correlation between FV size and fEPSPs is improved and similar to that of WT synapses. After LTP induction, lower FV amplitudes cause larger evoked responses. Notably, after LTP induction, there is a clear reduction in the basal FV amplitude of WT. Because the J20 mice were treated months before we performed the experiments, we hypothesized that ANDRO altered some of the presynaptic mechanisms with the consequent increase in the efficiency of the synapse.

Additional electrophysiological studies provided some clues regarding the mechanisms involved (Figure 7). The PPF protocol shows the evoked response to successive pulses separated by different time intervals (Figure 7B). PPF is a form of short-term plasticity and reflects the probability of release from presynaptic sites (Debanne et al., 1996; Jackman and Regehr, 2017). We demonstrated that J20 Tg mice have deficient PPF compared to WT and J20+ANDRO mice over a wide range of intervals. Moreover, the PPF index, comparing the basal PPF (pre-TBS) and after LTP induction (post-TBS), was also deficient during LTP studies. Thus, if there is a presynaptic component during the expression of LTP, both pre- and postsynaptic mechanisms should interfere (Schulz et al., 1994). We measured this interference (Figure 7E), which has been called attenuation (Wang and Kelly, 1997). A reduced degree of attenuation was found in J20 Tg mice, suggesting reduced participation of the presynaptic component during LTP. Instead, ANDRO-treated animals showed similar levels to WT mice. These data have several implications. One implication is that LTP appears to be less affected in this mutant than in other Tg mouse models of AD (Gengler et al., 2010; Serrano et al., 2014). We can report that, at the time we performed these experiments, LTP, which is regulated postsynaptically (Glanzman, 1994; Malenka and Bear, 2004), is significantly lesser in J20 mice compared to WT. The amount of attenuation is significantly different from those in the other two groups, which implies that the influence of presynaptic terminals is stronger in WT and ANDRO-treated mice than in J20 Tg mice. Therefore, we could infer that presynaptic failure was one of the first signs to be manifested in the J20 Tg model and might account for early markers of the disease, at least at the time we performed the experiments (7 months). We can predict that those failures will reach postsynaptic components later in life causing more severe deficiencies in LTP, or even its complete suppression, as occurs in the more severe double Tg model mice (Gengler et al., 2010; Serrano et al., 2014). The origin of this mechanism has been controversial, but there is strong evidence attributing it to postsynaptic activity dependent on Ca^2+^ and Ca^2+^-calmodulin (CaM) signaling and to inhibition of calcineurin (CaN) activity (Wang and Kelly, 1997). We did not explore these dependencies in our experiments.

In summary, we present evidence that ANDRO restores several aspects of neurophysiology including glucose metabolism, cognitive performance and synaptic transmission. ANDRO normalized the synaptic mechanisms acting on the presynaptic side to avoid failures in the output signals. An important step forward from here would be to demonstrate that presymptomatic treatment with ANDRO is able to delay the principal symptoms of AD, including those that concern glucose utilization, synaptic failure and the cognitive task. These findings suggest that a preventive treatment against AD could be feasible.

In future research, we would like to go a step further and test the preventive effect of ANDRO in more complex models of AD and, ultimately, to test this treatment in patients with AD or even in patients with early clinical manifestations of AD. Overall, we propose ANDRO as a feasible therapeutic drug to delay the early- and late-onset manifestations of neurodegenerative diseases such as AD.

## Author Contributions

PC and NI conceived and designed the experiments. PC, CO, DB, and VT performed the experiments. PC, CO, and VT analyzed the data. NI contributed to the reagents, materials, and analysis tools. PC, CO, VT, and NI wrote the manuscript. All authors read and approved the final manuscript.

## Conflict of Interest Statement

The authors declare that the research was conducted in the absence of any commercial or financial relationships that could be construed as a potential conflict of interest.
